# Hydrophobic Ag-Containing Polyoctylmethylsiloxane-Based Membranes for Ethylene/Ethane Separation in Gas-Liquid Membrane Contactor

**DOI:** 10.3390/polym14081625

**Published:** 2022-04-18

**Authors:** Evgenia Grushevenko, Alexey Balynin, Ruslan Ashimov, Stepan Sokolov, Sergey Legkov, Galina Bondarenko, Ilya Borisov, Morteza Sadeghi, Stepan Bazhenov, Alexey Volkov

**Affiliations:** 1 A.V. Topchiev Institute of Petrochemical Synthesis RAS, 119991 Moscow, Russia; ab@ips.ac.ru (A.B.); ashimov999@gmail.com (R.A.); sokolovste@ips.ac.ru (S.S.); legkov@ips.ac.ru (S.L.); bond@ips.ac.ru (G.B.); boril@ips.ac.ru (I.B.); sbazhenov@ips.ac.ru (S.B.); avolkov@ips.ac.ru (A.V.); 2 Department of Gas Chemistry, Faculty of Chemical Technology and Ecology, National University of Oil and Gas “Gubkin University”, 119991 Moscow, Russia; 3 Department of Chemical Engineering, Isfahan University of Technology, Isfahan 8415683111, Iran; m-sadeghi@iut.ac.ir

**Keywords:** membrane contactor, ethane/ethylene separation, polyoctylmethylsiloxane, Ag-containing membrane, polysiloxane membrane, composite membrane

## Abstract

The application of gas-liquid membrane contactors for ethane-ethylene separation seems to offer a good alternative to conventional energy-intensive processes. This work aims to develop new hydrophobic composite membranes with active ethylene carriers and to demonstrate their potential for ethylene/ethane separation in gas-liquid membrane contactors. For the first time, hybrid membrane materials based on polyoctylmethylsiloxane (POMS) and silver tetrafluoroborate, with a Si:Ag ratio of 10:0.11 and 10:2.2, have been obtained. This technique allowed us to obtain POMS-based membranes with silver nanoparticles (8 nm), which are dispersed in the polymer matrix. The dispersion of silver in the POMS matrix is confirmed by the data IR-spectroscopy, wide-angle X-ray diffraction, and X-ray fluorescence analyses. These membranes combine the hydrophobicity of POMS and the selectivity of silver ions toward ethylene. It was shown that ethylene sorption at 600 mbar rises from 0.89 cm^3^(STP)/g to 3.212 cm^3^(STP)/g with an increase of Ag content in POMS from 0 to 9 wt%. Moreover, the membrane acquires an increased sorption affinity for ethylene. The ethylene/ethane sorption selectivity of POMS is 0.64; for the membrane with 9 wt% silver nanoparticles, the ethylene/ethane sorption selectivity was 2.46. Based on the hybrid material, POMS-Ag, composite membranes were developed on a polyvinylidene fluoride (PVDF) porous support, with a selective layer thickness of 5–10 µm. The transport properties of the membranes were studied by separating a binary mixture of ethylene/ethane at 20/80% vol. It has been shown that the addition of silver nanoparticles to the POMS matrix leads to a decrease in the ethylene permeability, but ethylene/ethane selectivity increases from 0.9 (POMS) to 1.3 (9 wt% Ag). It was noted that when the POMS-Ag membrane is exposed to the gas mixture flow for 3 h, the selectivity increases to 1.3 (0.5 wt% Ag) and 2.3 (9 wt% Ag) due to an increase in ethylene permeability. Testing of the obtained membranes in a gas-liquid contactor showed that the introduction of silver into the POMS matrix makes it possible to intensify the process of ethylene mass transfer by more than 1.5 times.

## 1. Introduction

The separation of saturated and unsaturated hydrocarbons is one of the most important tasks in the petrochemical industry, since ethylene and propylene are the main products of organic synthesis (~160 and ~100 million tons/year, respectively), being precursors for a broad range of widely used chemical compounds. The main difficulty in separating olefins and paraffins with the same number of carbon atoms is the small difference in their boiling points. This leads to high capital and energy costs in the traditional separation technology–low-temperature distillation method at elevated pressures, where metal-intensive distillation columns with more than 100 plates are used, commonly known as ethylene and propylene fractionators or C2 and C3 splitters [1].

An alternative approach to the problem is the chemical absorption of olefins in gas-liquid membrane contactors, which combines the advantages of the absorption separation (process continuity and the high selectivity of absorption of olefins by aqueous solutions of transition metal salts) and the advantages of membrane technologies (known and constant mass transfer area, compactness due to the high specific active area, modularity, the absence of direct phase mixing, wide range, and independent control of gas and liquid flows). The separation is ensured by the different solubilities of saturated and unsaturated hydrocarbons in the absorption liquid [1,2]. Solutions of the complexing agents (silver [3,4,5,6] or copper [7] salts) are used as absorbents, while most previous studies are devoted to the removal of olefins using solutions of silver nitrates [3,4,5,6,8,9] and tetrafluoroborates [10] in water or ionic liquids [10,11]. In the case of the closed-loop process of unsaturated hydrocarbons absorption/desorption using membrane contactors, olefin-selective composite membranes are the most promising possibility [1,11,12,13,14]. Their thin non-porous layers provide the facilitated transport of olefins and, at the same time, serve as a wetting barrier, preventing contact of the absorbent with the porous structure of the membrane support. The idea of using such membranes is based on the works of I. Bekman et al. [8,15], which argue that in a closed system consisting of a membrane absorber and a desorber, two extreme situations could occur:

(1) At low absorbent flow rates, the selectivity of the process is determined by the selectivity of the liquid (which is high) and, in this case, the overall olefin flux is low due to the insufficient supply of absorbent.
(2) At high absorbent flow rates, this closed system acts as a simple membrane gas separation module (the overall olefin flux is high, but the process selectivity is determined by the selective properties of the polymer membrane, which is often lower than that for the absorbent).

Therefore, the implementation of olefin-selective composite membranes maximizes the selectivity and productivity of the membrane-contactor system in the entire range of absorbent flows. Since the pioneering work of Nymeijer et al., published in the early 2000s [16,17,18], no studies in this direction have been conducted, while the development and creation of the next generation of high-performance composite olefin-selective membranes for gas-liquid contactors are extremely relevant and important.

Membrane technologies are promising for various separation tasks in oil refining and petrochemical processes. In particular, they have the potential for a breakthrough in the separation of saturated and unsaturated hydrocarbons [19]. The most widely used and thoroughly investigated method in this field is membrane gas separation, which utilizes both porous and non-porous membranes. In the first case, membranes based on carbon molecular sieves (CMS) [20], which, due to their fine porous structure (ultra- and micropores), separate olefins and paraffins due to the difference in the size of their molecules, are considered advanced. W. Koros’s and I. Pinnau’s research groups showed that carbon membranes with the desired properties can be produced by the controlled pyrolysis of polymer precursors [21,22,23]. However, the use of such membranes is still limited due to their relatively low mechanical characteristics and the difficulty of scaling their production [24,25]. On the contrary, non-porous polymer membranes, where separation is achieved due to the difference in the sorption and diffusion rates of olefin and paraffin molecules in the membrane materials, lack these disadvantages. Moreover, membranes based on cellulose, polyphenylene oxide, siloxanes, polymers with intrinsic microporosity, etc. were studied and reviewed in [26]. Polyimides are also proposed for use in this task [27,28]; however, the ideal separation selectivity in such membranes is low and does not exceed 5 for the ethylene/ethane pair, and is less than 70 for the propylene/propane pair [26].

To increase separation selectivity, new materials have been developed. They contain active components (for example, silver or copper ions) in the polymer or (in the case of hybrid membranes) in the filler structure that selectively interact with olefin molecules to form π-complexes and conduct the so-called facilitated olefin transport mechanism [29,30,31,32,33]. However, such membranes lose their properties due to the degradation/deactivation of active components in the membrane structure over time [34,35]. In addition, the transport properties of such membranes are often high when moisture is present in the membrane matrix, due to humid gas mixture separations [32,36,37]. On the other hand, an anhydrous medium is required in most olefin and paraffin treatment processes. An alternative is the so-called liquid membranes, in which the active complexing agent in the liquid phase is immobilized in the pores of the support membrane. Aqueous solutions of silver and copper salts [38], deep eutectic solvents [39], and ionic liquids [40,41] are used for these membranes. However, these liquid-immobilized membranes are also unstable due to the drying of the liquid phase over time, the deactivation of the carrier, and the displacement of the liquid phase from the pores of the support membrane [35].

Usually, the use of porous hollow-fiber membranes is preferred to reduce mass transfer resistance in the membrane contactor. However, this configuration has a significant drawback because the membrane pores can be gradually wetted and filled with absorption liquid until the phases are completely mixed, which leads to an increase in mass transfer resistance [42,43]. This phenomenon is also characteristic of the membrane absorption separation of olefins and paraffins [44]. Several techniques have been proposed to prevent this:

(1) The use of membranes made of hydrophobic materials. For example, polyvinylidene fluoride (PVDF) membranes were proposed in [4], but even such hydrophobic membranes are susceptible to wetting [44]. The most stable membranes are from polytetrafluoroethylene (PTFE) [44], but they are more complex and expensive to produce [14].
(2) Surface modification of the membranes to impart hydrophobic properties. For example, the authors of [44] reported the stable total mass transfer coefficient for 2 months in the separation of a propane/propylene mixture with a contactor, based on ceramic hollow-fiber membranes from Al_2_O_3_ modified with 1H,1H,2H,2H-Perfluorooctyltriethoxysilane. A method using the surface hydrophobization of hollow-fiber polysulfone membranes with commercial perfluorinated acrylic copolymer (PFAC) ProtectGuard was proposed by the authors of [9].
(3) The use of composite membranes with a thin non-porous layer. This thin protective layer should have high olefin permeability, be located on the side of the liquid phase, and prevent the filling of the pores of the support membrane with a liquid absorbent [13].

The latter approach seems to be the most promising since it solves the problem of membrane wetting and significantly expands the operating pressure range of the membrane-absorption system [42]. In addition, the correct choice of material for the selective layer allows the production of olefin-selective membranes that radically improve the properties of the system, achieving high olefin fluxes with high hydrocarbon separation factors. Such an approach was first proposed by Nymeijer et al. [13,16,17,18]. They used polymers that are wetted by the absorbent and swell in it as materials for the barrier layer. The purpose of this was the impregnation and retention of silver ions in the polymer matrix and the prevention of the membrane from drying out or deactivating the active component. For example, composite membranes based on polypropylene (PP) hollow-fiber support, with a selective layer of ethylene propylene diene terpolymer (EPDM) with a thickness of 8 μm, were used to separate the ethylene–ethane mixture [17]. Membranes with a selective layer (10 μm) of sulfonated polyester ether ketone (sPEEK) on PP supports were proposed by the authors of [18]. Due to the electrostatic attraction between the negatively charged sPEEK sulfo-groups and positively charged Ag^+^ ions in the absorbent, enormous ethylene/ethane separation factors (>2700) were achieved. Finally, in continuation of sPEEK-PP membranes studies, the productivity of the membrane-absorption system for ethylene was increased by 2–5 times, while maintaining high selectivity for ethylene (separation factor > 150) [16]. This was achieved using composite membranes with a thin layer of a copolymer of poly(ethylene oxide) (PEO) and poly(butylene terephthalate) (PBT) with a thickness of 24.3 μm. Silver nitrate aqueous solutions were used as the absorbent in all these studies, and the contactors showed stable mass transfer characteristics for more than 4 weeks of experiments.

The aim of this work is to develop a new type of olefin-selective composite membrane for the separation of saturated and unsaturated hydrocarbons by the membrane-absorption technique, using gas-liquid contactors. Its selective layers will be composed of hydrophobic swollen polymer systems, with the active components (silver ions) impregnated in the polymer matrix. Such membranes will provide high selectivity and unsaturated hydrocarbon flux, due to the facilitated transport of olefins. For the first time, polyoctylmethylsiloxane (POMS) will be used as a hydrophobic matrix.

## 2. Materials and Methods

### 2.1. Compounds

Polymethylhydrosiloxane (PMHS) with a number-average molecular weight Mn = 1900 g/mol (ABCR, Karlsruhe, Germany), a Carsted catalyst–platinum complex of 1,3-divinyl-1,1,3,3-tetramethyldisiloxane in xylene (~2% Pt, Sigma-Aldrich, St. Louis, MO, USA), 1-octene (98%, Sigma-Aldrich, St. Louis, MO, USA), 1,7-octadiene (98%, Sigma-Aldrich, St. Louis, MO, USA), polydimethylsiloxane with a number-average molecular weight Mn = 25,000 g/mol (Sigma-Aldrich, St. Louis, MO, USA), silver tetrafluoroborate (98%, Sigma-Aldrich, St. Louis, MO, USA), silver nitrate (95%, Podolsk, Chimmed, Russia), isooctane (99%, Podolsk, Chimmed, Russia) were used without further purification for membrane preparation.

### 2.2. Membrane Preparation

**Figure 1:**
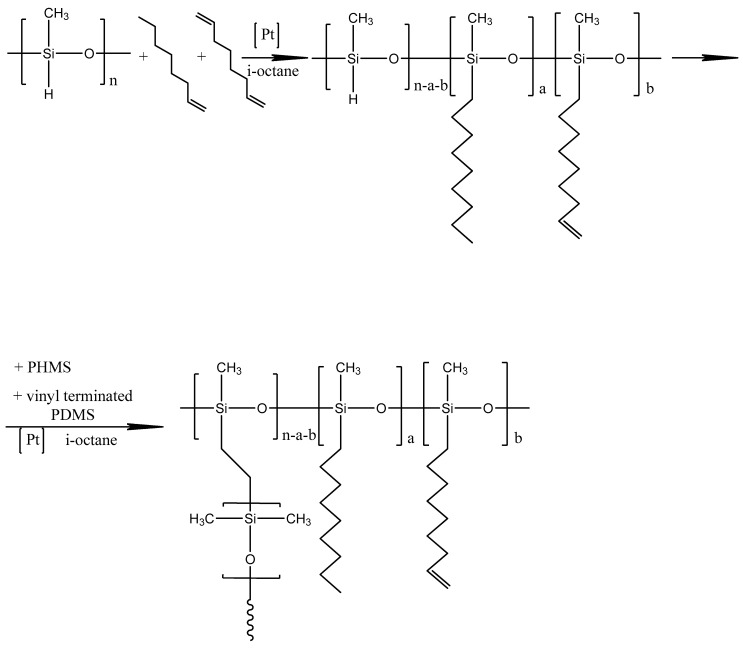
Scheme of POMS synthesis.

POMS-based membranes with POMS-Ag and without Ag-containing POMS were prepared according to the following method, developed based on earlier studies [45,46,47,48]. To begin, PHMS, 1-octene, and 1,7-octadiene in mole relation to 10:9.4:2.6 were mixed in the presence of 2.3 wt% Carsted catalyst (to pure reactive) in the isooctane media. The resulting solution was stirred at a temperature of 60 °C for 2 h. After that, the PDMS dissolved in isooctane was added to the reaction mixture. The stirring at 60 °C was continuous for 1 h. At the end of this time, a solution of PMHS in isooctane (8 wt% of the initial amount of PMHS) was added to the reaction mixture. Subsequently, stirring continued until the viscosity of the solution reached 13 MPa s. The resulting solution was used to obtain the POMS membranes. A scheme of POMS synthesis is represented in Figure 1. To obtain POMS-Ag membranes, dry AgBF_4_ was added to the reaction mixture and stirred in an ultrasonic bath (Sapfir, Moscow, Russia) for 20 min. To obtain a dense film membrane result, the solution was cast on a Teflon surface with subsequent drying at 100 °C for 24 h. The residual solvent was removed by drying in the vacuum chamber. For obtaining POMS-based composite membranes, we used a flat-sheet porous membrane: microfiltration PVDF-based membrane MFFK-1 (Vladipor, Vladimir, Russia). Composite membranes were prepared using the kiss-coating method described in detail in [48,49]. After the coating procedure, the composite membranes were dried at 100 °C for 12 h.

### 2.3. Scanning Electron Microscopy (SEM)

Scanning electron microscopy (SEM) was used to characterize the structure and morphology of the membranes. SEM was carried out on a Thermo Fisher Phenom XL G2 Desktop SEM (Waltham, MA USA). Cross-sections of the membranes were obtained in liquid nitrogen after preliminary impregnation of the specimens in isopropanol. A thin (5–10 nm) gold layer was deposited on the prepared samples in a vacuum chamber (≈0.01 mbar) using a desktop magnetron sputter, the Cressington 108 Auto Sputter Coater (Watford, UK). The accelerating voltage during image acquisition was 15 kV. Further image analysis and the determination of the selective layer thickness were carried out using the Gwyddion software (ver. 2.53).

### 2.4. Porosimetry

Analysis of the support transport pores was performed with the liquid-liquid displacement technique using the Poroliq 1000ML (IB-FT GmbH, Berlin, Germany), with isobutanol and water as the wetting and non-wetting liquids, respectively.

### 2.5. Wide-Angle X-ray Scattering (WAXS)

The X-ray diffraction spectra were obtained using a Rigaku Rotaflex RU-200 (Tokyo, Japan) with a rotating copper anode (Cu Kα, emission; Ni, filter) at apparatus operating conditions 30 kV–100 mA. X-ray photography was performed using a horizontal wide-angle goniometer, the Rigaku D/MAX-RC (Tokyo, Japan), using the Bragg–Brentano scheme in θ–2θ geometry. Scanning was carried out at a 5–55° angle range at 2θ, with a speed of 2°/min and 0.04° increment. A scintillation counter was used as a detector of the diffracted X-ray emission. Measurements were taken at 20 °C and at −190 °C. A specialized low-temperature attachment was used to obtain a diffraction pattern of the specimen, cooled down to liquid nitrogen temperature (−196 °C). Membrane specimens with a thickness of 500 μm were mounted to a vertical copper table. The diffraction patterns that were obtained were processed using the Fityk program (by Marcin Wojdyr, Poland): background noise was subtracted, and diffraction patterns were approximated using the deconvolution technique by the sum of several Gaussian peaks [46].

### 2.6. Infrared Spectroscopy

IR spectra registration was carried out in the film’s surface reflection mode using an IR microscope, HYPERION-2000, conjugated with an IR-Fourier spectrometer, IFS 66 v/s Bruker (Elmsford, NY, USA) (Ge crystal, scan – 50, resolution 2 cm^−1^, range 600–4000 cm^−1^).

### 2.7. Sorption Measurement

The pure gas and vapor sorption isotherms were measured using a XEMIS gravimetric sorption analyzer (Hiden Isochema, Warrington, UK) with buoyancy correction at 35 °C under gas pressure up to 1 atm and water vapor pressure up to 30 mbar (p/p 0 ≈ 0.94). At the corresponding pressures, the difference between the absolute and excess adsorption amounts is insignificant [50], so no additional models were used to determine the absolute adsorption. The skeletal densities of the samples were determined using the helium calibration method, as described in [50]. The density of vapors and gases was obtained using the NIST REFPROP V.9.1 database. The measured skeletal densities were (1.28 ± 0.03) g/cm^3^ and (1.14 ± 0.05) g/cm^3^ for the POMS and POMS-Ag samples, respectively. Before starting the sorption measurements, the samples were evacuated at 100 °C for 5 h to remove the solvent residues. Each adsorption isotherm was measured at least 3 times and, between identical measurements, the sample was evacuated for 2 h at 45 °C.

### 2.8. X-ray Fluorescence Analysis

The elemental composition of the POMS and POMS-Ag membranes was determined using wavelength-dispersive X-ray fluorescence analysis, carried out on an ARL PERFORM’X (Thermo Fischer Scientific, Waltham, MA USA) sequential X-ray fluorescence spectrometer using a rhodium tube [51]. Up to 79 elements were analyzed and the percentage composition of the sample was calculated using the UniQuant program, to a relative error of 5%.

### 2.9. Gas Permeation Measurements

The POMS and POMS-Ag composite membrane gas transport properties were studied in the process of separating a mixture of ethylene-ethane gas. The gas mixture was prepared with Moscow gas processing plant (Moscow, Russia). The ethylene/ethane ratio was 20% vol./80% vol. The gas permeability of the flat-sheet membranes was studied, employing the apparatus described in detail in a previous study [47], with the following conditions: temperature, 23.6–25 °C; effective membrane permeation area, 13.8 cm^2^; downstream membrane pressure, 2–5 cm/Hg; stage-cut, 2–5%. A feed, permeate and retentate analysis was conducted using a gas chromatograph, the Gazokhrom-2000 (Chromatek, Yoshkar-ola, Russia), with a column filled with 20% heptadecane filler on the diatomite carrier. Analysis of a 0.5 mL gas sample was carried out under the following conditions: the dosing valve’s thermostat temperature was 50 °C, the column’s thermostat temperature was 50 °C, the detector’s thermostat temperature was 160 °C, and the flow rate of carrier gas (helium) was 30 mL/min. The measurement error is no higher than 7%. The permeance of the gas mixture component (*P*/*l*, m^3^/(m^2^ atm h)) of the membranes was calculated using Equation (1):

(1) $$P/l=\frac{C_{i}^{perm}\cdot J^{perm}}{S\cdot \left(C_{i}^{feed}\cdot p^{feed}-C_{i}^{perm}\cdot p^{perm}\right)}$$

where *C_i_* is the *i*-concentration in the gas mixture, *p* is pressure, *J^perm^* is the permeate flux, *S* is the membrane surface area.

The selectivity of separation of the mixture components *i* and *j* was calculated using Equation (2):

(2) $$\alpha_{mix}=\frac{P/l_{i}}{P/l_{j}}$$

### 2.10. Membrane Contactor Gas-Liquid

**Figure 2:**
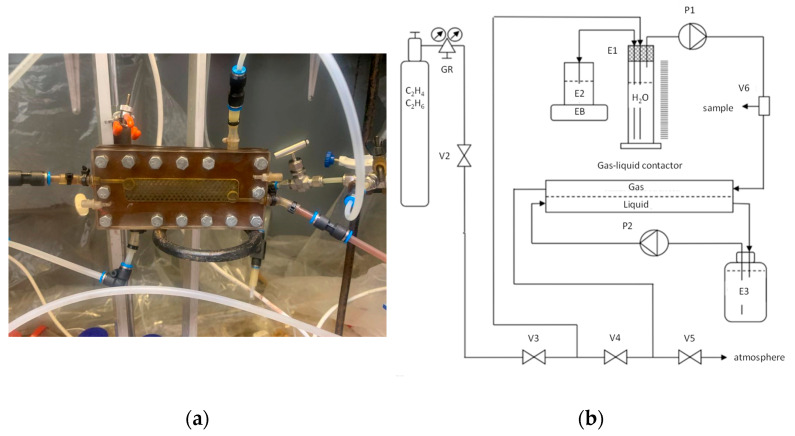
General view of a flat-frame membrane gas-liquid contactor (**a**) and overall scheme (**b**).

A study of the separation of the ethylene-ethane binary mixture in the process of membrane absorption was carried out with a laboratory gas-liquid membrane contactor. The membrane area in the flat frame modules was 20 cm^2^. A general view of the module is shown in Figure 2a. The scheme of the laboratory setup for studying the separation of ethylene and ethane mixtures in a laboratory gas-liquid membrane contactor, based on the modules developed in this work, is similar to that presented in Figure 2b and described elsewhere [52].

The inlet flow of the gas mixture was supplied from the side of the porous substrate without excess pressure (the pressure of the gas mixture was at the level of 1 atm). From the side of the selective layer, a liquid absorbent, an aqueous solution of silver nitrate, was supplied using an LS-301 peristaltic pump (LOIP, Sait-Peterburg, Russia). For the flat-frame module, the silver nitrate solution was supplied from the side of the selective layer. A liquid absorbent with a silver nitrate concentration of 3.5 M and a gas mixture were fed in using the recirculation mode. The preparation comprising aqueous solutions of silver nitrate was carried out by dissolving the required sample of salt in distilled water. Samples for analysis were taken every hour. The absorbent liquid was circulated continuously in the liquid circuit at a predetermined rate. The analysis of the composition of the gas mixture was carried out with a Gazokhrom-2000 gas chromatograph (Khromatek, Yoshkar-ola, Russia).

The molar flow of the gas mixture component (L/(m^2^h)) in the contactor was determined using Equation (3):

(3) $$J_{i}=\frac{V_{i}\left(0\right)-V_{i}\left(t\right)}{t\;\cdot A}\frac{273}{T\vartheta_{0}}$$

where *T* is the temperature of the experiment, *t* is the estimated time of the experiment, *V_i_* (0) is the initial volume of gas, *V_i_* (*t*) is the volume of gas at time *t*. *A* is the area of the interface equal to: *A* = NπdL, where N is the number of fibers in the membrane contactor module, d is the outer diameter of the fiber, and L is the length of the fibers in the module.

The permeability of the gas mixture component was calculated as the ratio of the gas mixture component flow to the transmembrane difference in partial gas pressures. The mass transfer coefficient in the gas phase K (cm/s) can be calculated using Equation (4):

(4) $$K_{i}=\frac{J_{i}}{\bar{\Delta x_{i}}}\frac{RT}{p}$$

where *J_i_* is the molar flow of gaseous ethylene, determined by Equation (3), $\bar{\Delta x_{i}}$ is the mean logarithmic difference between ethylene concentrations in the gas mixture being separated and in the permeate, *R* is the universal gas constant, and *p* is the total gas pressure in the mixture being separated.

Since the liquid absorbent is in excess, the gas concentration in the liquid can be ignored [50]. Then, the expression for $\bar{\Delta x_{i}}$ corresponds to Equation (5):

(5) $$\bar{\Delta x_{i}}=\frac{x_{i}\left(0\right)-x_{i}\left(t\right)}{\ln\left(x_{i}\left(0\right)/x_{i}\left(t\right)\right)}$$

where *x_i_* (0) is the initial molar concentration of the gas mixture component at the inlet to the membrane contactor, and *x_i_* (*t*) is the molar concentration of the gas mixture component after it has been in the contactor for a period of time *t*.

The selectivity value of ethylene/ethane separation in a membrane contactor is determined by Equation (6):

(6) $$\alpha =K_{C2H4}/K_{C2H6}$$

## 3. Results and Discussion

### 3.1. Choice of Ethylene-Selective Additive and Its Concentration

**Figure 3:**
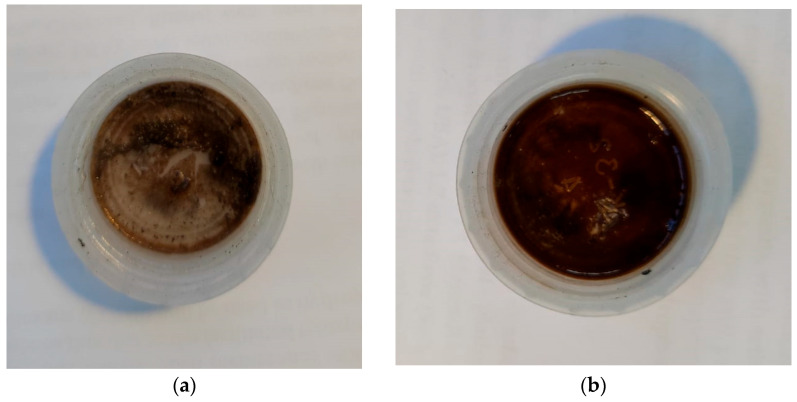
Types of POMS polymer films with the addition of a silver salt: (**a**) AgNO_3_, (**b**) AgBF_4_.

Silver nitrate (AgNO_3_) and silver tetrafluoroborate (AgBF_4_) were chosen as the active components. In the course of the project, for the first time, a technique was developed for the in-situ production of materials and membranes, based on silver-filled POMS. The addition of the active component (silver) to the polymer gel was carried out after the gel had reached the viscosity required for membrane formation. Figure 3 shows a comparison of the appearance of the obtained silver salt dispersion in POMS: AgNO_3_, (a), AgBF_4_, (b). It should be noted that the dispersion obtained for AgBF_4_ is more uniform (Figure 2b), while in the sample with AgNO_3,_ there is a clear agglomeration of particles. Based on the results obtained, AgBF_4_ was chosen for further experiments.

**Figure 4:**
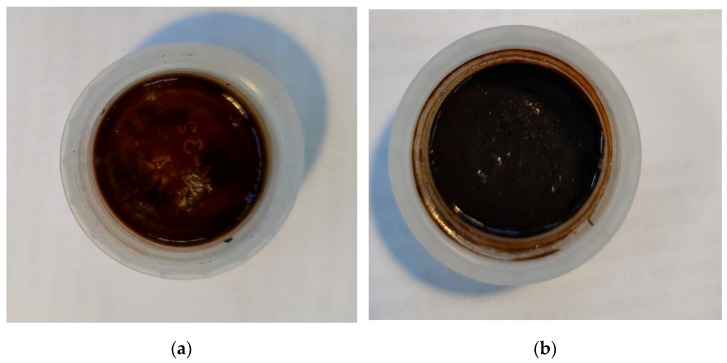
Types of POMS polymer films with the addition of AgBF_4_: (**a**) 1 wt%, (**b**) 20 wt%.

POMS films with different AgBF_4_ contents were obtained from 1 to 50 wt%. When the AgBF_4_ concentration in the polymer was more than 20 wt%, the formation of a mechanically stable film did not occur. In that case, the color intensity of the polymer film increased during the transition from 1 to 2 wt% AgBF_4_ (Figure 4). At the same time, the uniformity of staining was maintained.

To select the composition of the membrane material to obtain composite membranes used in the separation of ethylene and ethane in gas-liquid membrane contactors, in samples with 1.8 wt% and 20 wt% AgBF_4_. These concentrations were chosen because of the film-forming ability of the polymer and the convenience of sample preparation.

According to elemental analysis, the silver content in the obtained polymer samples was determined (Table 1). Thus, in the case of the original POMS, the presence of silver was not recorded. In the case of the POMS sample with the addition of 1.8% AgBF_4_ (without pre-drying the salt), the silver content was determined to be 0.5 wt% (sample symbol, POMS-Ag1). In the case of a sample with 20 wt%, AgBF_4_ recorded 9.4 wt% silver in the sample (sample symbol, POMS-Ag9). The Si:Ag ratio was 10:0.11 for POMS-Ag1 and 10:2.2 for POMS-Ag9. The decrease in the silver content relative to the expected value (based on the ratio of the weight of the added AgBF4 and the weight of the polymer) is associated with the high hygroscopicity of the salt (the real weight of silver is lower; the contribution of the water is not considered).

To determine the form of silver in the POMS polymer matrix, IR spectra and diffraction patterns were taken.

**Figure 5:**
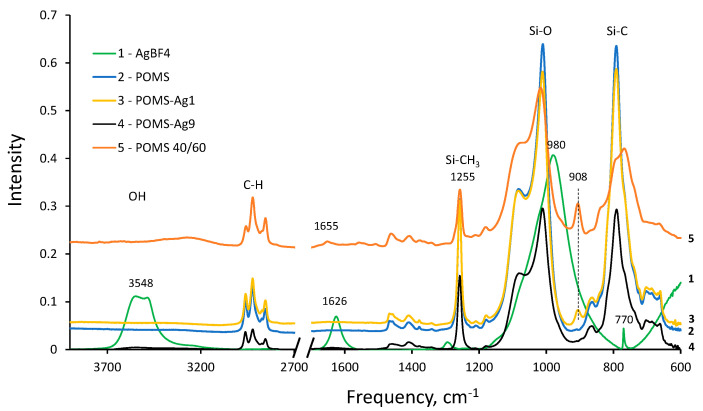
FTIR-spectra: 1, AgBF_4_; 2, POMS; 3, POMS-Ag1; 4, POMS-Ag9; 5, POMS 40:60. Adapted with permission from Ref. [45]. Copyright 2017, Pleiades Publishing, Ltd.

Figure 5 presents the FTIR spectra of samples POMS (2), POMS-Ag1 (3), and POMS-Ag9 (4) in comparison with the spectrum of crystalline AgBF_4_ (1). The spectrum of crystalline AgBF_4_ contains a very intense band at 980 cm^−1^ from the stretching vibrations of B-F bonds and a weak band at 770 cm^−1^, which practically coincide with the bands from Si-O and Si-C in the POMS spectra and can appear in the spectrum POMS-Ag only at a sufficiently high content of AgBF_4_ in the mixture with siloxane. Two rather intense bands in the spectrum of AgBF_4_ (1), 3548 and 1626 cm^−1^, refer to the vibrations of water with crystallization in the composition of silver salt crystals and cannot appear in the spectrum of the salt dissolved in isooctane. In spectra 3 and 4 (POMS + AgBF_4_), there are no spectral features of the silver salt. The appearance of a new band in the spectrum at 908 cm^−1^ is apparently due to inhomogeneity in the composition of the links in the POMS used. This band manifested itself in previously recorded POMS samples of various compositions and was attributed to the terminal vinyl group in 1,7-octadiene that was attached to the siloxane by one vinyl group [45]. In POMS (60:40 spectrum 5), this band has a rather high intensity and, besides, the second (weaker) band of the terminal vinyl group from C=C stretching vibrations at 1655 cm^−1^ appears in the spectrum. In spectrum 3 (Figure 5), the 908 cm^−1^ band has a low intensity, i.e., the proportion of such terminal vinyl groups in the polymer composition does not exceed 1% (mol), and in spectra 2 and 4, the vinyl groups do not appear.

**Figure 6:**
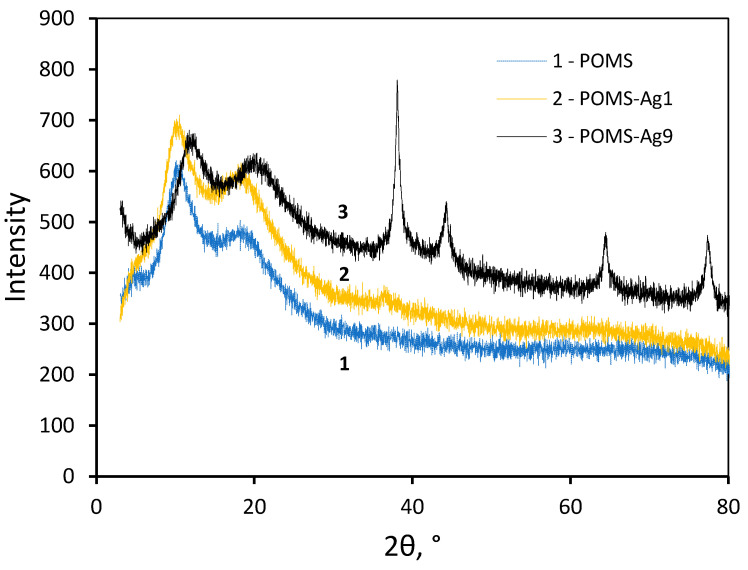
WAXD data: 1, POMS; 2, POMS-Ag1; 3, POMS-Ag9.

According to X-ray phase analysis, a diffraction pattern is observed in the POMS-Ag9 sample, demonstrating the presence of a pure silver phase with a crystallite size of about 8 nm (Figure 6). For the POMS-Ag1 sample, there are no clear signs of a pure silver phase. Apparently, the concentration of silver in the sample is insufficient to form a clear diffraction pattern.

Based on the data obtained, it can be concluded that the proposed method of in-situ membrane preparation allowed us to obtain MMMs with a relatively uniform dispersion of silver nanoparticles in the POMS polymer matrix. Silver nanoparticles can improve the selectivity of polymer membranes for ethylene, while the authors of [53] note that hybrid membranes with silver nanoparticles have more stable transport characteristics than membranes containing silver ions in the chemical structure.

### 3.2. Sorption and Gas Transport Properties

To determine the ability to selectively transport ethylene, the sorption capacity of the obtained membrane materials for saturated and unsaturated hydrocarbons was studied for the first time using the example of ethylene and ethane. Water vapor sorption was also studied, which is important for understanding the transport of absorbent vapors in the implementation of the membrane absorption separation of olefins and paraffins in gas-liquid contactors. In terms of the materials under study, the selectivity of ethylene transfer through the membrane would be determined precisely by the sorption component. This is for two reasons: (1) in POMS, the contribution of the sorption component to transport is the determining factor in the high permeability of this material for hydrocarbons; (2) silver particles play the role of ethylene sorption centers, which should lead to an intensification of its transfer through the membrane.

**Figure 7:**
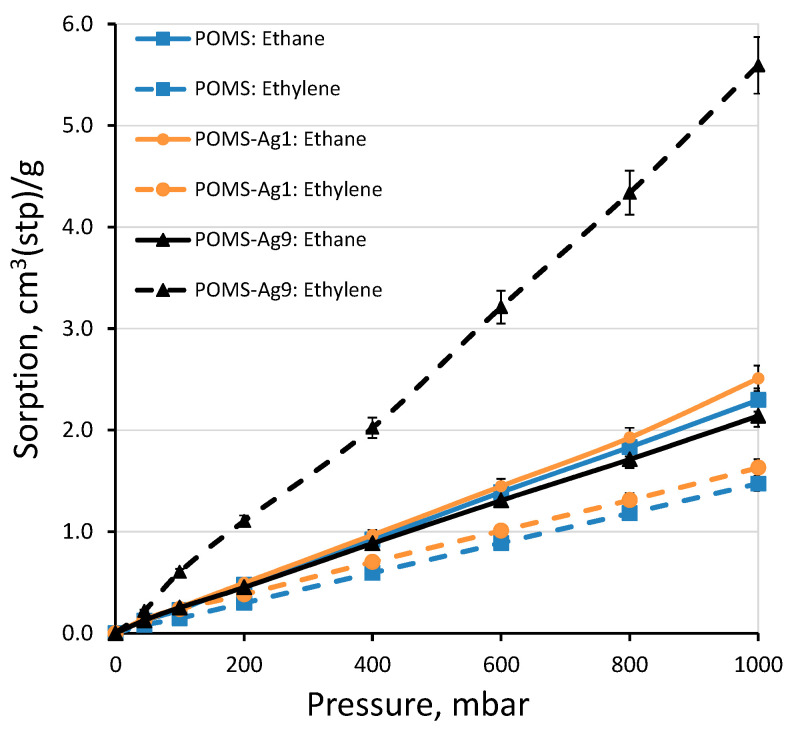
Sorption isotherms of ethane (solid line) and ethylene (dashed line) in POMS (square), POMS-Ag1 (circle), and POMS-Ag9 (triangle) at 35 °C.

In all the studied polymers, with an increase in the pressure of the gas, the sorption value increased (Figure 7). For the initial POMS material, the sorption of ethane was predictably higher than the sorption of ethylene, which means an increased selectivity of membrane transport for ethane since the sorption component of transport is decisive for polysiloxanes [54]. When silver particles were introduced into the POMS polymer matrix, the picture changed. The introduction of 0.5 wt%. silver in the POMS-Ag1 sample led to an insignificant increase in the sorption of both ethane and ethylene. So, for example, at a pressure of 600 mbar, the sorption of ethylene and ethane in POMS was 0.889 ± 0.023 and 1.390 ± 0.011 cm^3^ (STP)/g, respectively. For POMS-Ag1, these values were 1.011 ± 0.012 and 1.4494 ± 0.003 cm^3^(STP)/g. With an increase in the amount of silver in POMS to 9 wt%, there was a fundamental change in the sorption of ethylene, with a slight decrease in the sorption of ethane. At 600 mbar, the sorption of ethylene in POMS-Ag9 increased to 3.212 ± 0.029 cm^3^(STP)/g.

A sharp increase in ethylene sorption with POMS-Ag9 compared to POMS-Ag1 and POMS correlates well with the diffraction pattern presented in Figure 6: it was only in the POMS-Ag9 sample that significant peaks of crystalline silver were observed, while in the POMS-Ag9 sample, POMS-Ag1, only a small peak was observed in the region of 38°.

If we consider the selectivity of the sorption of membrane materials for ethylene (the ratio of the sorption of ethylene to the sorption of ethane can be found in Table 2), then we note the following trends for the samples under study. In the case of pure POMS, no dependence of the sorption selectivity on the applied pressure was observed. This value was in the range of 0.64 ± 0.02. For a sample of POMS-Ag1 containing 0.5 wt% silver nanoparticles in the polymer, a decrease in selectivity was observed with increasing gas pressure: from 0.90 for 40 mbar to 0.65 for 1000 mbar. Apparently, such a decrease in the selectivity of sorption is due to the low selectivity of the polymer matrix and the small contribution of the silver nanoparticles, due to their small amount. For the POMS-Ag9 sample, the ethylene selectivity increases with increasing gas pressure from 1.73 for 45 mbar to 2.61 for 1000 mbar. This additionally indicates that the concentration of silver nanoparticles at the level of 9 wt% makes a significant contribution to the properties of the hybrid membrane material.

**Figure 8:**
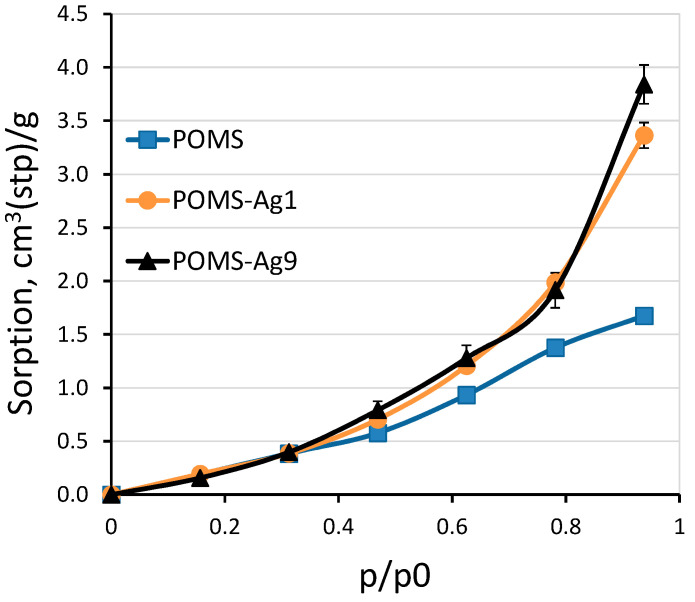
Water vapor isotherms in POMS (square), POMS-Ag1 (circle), and POMS-Ag9 (triangle) at 35 °C.

The water vapor sorption in POMS, POMS-Ag1, and POMS-Ag9 membrane materials was also considered (Figure 8). The introduction of silver into the POMS matrix led to an increase in the sorption of water vapor, which should affect the intensity of the transfer of absorbent vapor through the membrane. However, it should be noted that there was no significant difference in water sorption for POMS-Ag1 and POMS-Ag9 in the measured pressure range (from 5 to 30 mbar).

It can be concluded that 0.5 wt% silver in the POMS matrix may not be sufficient to elicit macro effects in the transport of ethylene and ethane through the membrane. When the concentration of silver nanoparticles was 9 wt%, this had a significant effect on sorption. This effect probably leads to a noticeable change in the ethylene transport through the membrane. To confirm these assumptions, the gas transport properties of these materials were studied.

**Figure 9:**
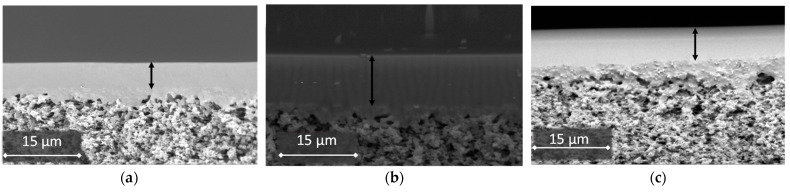
SEM images of samples of the flat composite membranes: (**a**) POMS/MFFK, 6 µm; (**b**) Ag1/MFFK, 10 µm; (**c**) Ag9/MFFK, 8 µm.

To study the gas transport properties of materials, flat-type composite membranes were obtained on a microfiltration support of MFFK-1 (Figure 9). Symbols of the obtained membranes: Ag1/MFFK, for POMS-Ag1 on the MFFK-1 support; Ag9/ MFFK, for POMS-Ag9 on the MFFK-1 support. The thickness of the selective membrane layer was: POMS/MFFK, 6 µm; Ag1/MFFK, 10 µm; Ag9/MFFK, 8 µm. The study of the effect of the addition of silver nanoparticles on the transport properties of membranes was carried out in the process of separating a binary mixture of 20% vol. ethylene/80% vol. ethane.

The membrane was exposed to the flow of an ethylene/ethane mixture for 3 h to determine the effect of the silver nanoparticles. Table 3 presents the transport and separating properties of the resulting composite membranes before and after exposure to a gas mixture. The POMS/MFFK membrane did not change its transport properties over time and retained an ethylene permeance of 0.35 m^3^/(m^2^atm h) and an ethylene/ethane pair selectivity of 0.85. This is consistent with the data on the sorption of pure gases in the membrane material. For polysiloxanes (POMS), the permeability coefficient was determined by the sorption component. In addition, the difference in the kinetic diameter of ethane (4.44 Å) and ethylene (4.163 Å) did not significantly affect the transport of gases through the membrane. When silver was added to the material, a decrease in membrane permeability was observed compared to POMS/MFFK. For Ag1/MFFK, the ethane and ethylene permeances were 0.20 and 0.20 m^3^/(m^2^ atm h), and for Ag9/MFFK, the ethane and ethylene permeances were 0.13 and 0.17 m^3^/(m^2^ atm h). If we calculate the permeance normalized to thickness (permeability coefficient) in the series POMS/MFFK, Ag1/MFFK, Ag9/MFFK, the ethane permeability coefficient decreases (6715, 5460, 2840 Barrer). The ethylene permeability coefficient also decreases in series (5730, 5460, 3710 Barrer). Such a change in transport properties is associated with a decrease in the diffusion component of the permeability. This decrease in permeability is associated not only with the difference in the selective layer thicknesses but also with an increase in the diffusion path for gas molecules. This is due to the filling of the polymer matrix with silver nanoparticles. It should be noted that the ethylene permeability coefficient increased after exposure of the membranes in the mixture to be separated for 3 h. It became higher for Ag1/MFFK (6280 Barrer) and Ag9/MFFK (6550 Barrer) membranes than for POMS/MFFK membranes.

The selectivity of separation by the ethylene/ethane gas pair increased with the introduction of silver nanoparticles into the POMS matrix (Table 3). Thus, the original POMS/MFFK membrane did not have selectivity for ethylene (0.85). At the same time, the introduction of only 0.5 wt% Ag made it possible to obtain a membrane that did not have selective permeability to both ethane and ethylene (Ag1/MFFK, 1.0). The introduction of 9 wt% nanoparticles made it possible to obtain a membrane that was selective for ethylene (Ag9/MFFK, 1.31). It is important to note that exposure of the membrane to the current of the binary mixture being separated for 3 h led to a significant increase in selectivity for both the Ag1/MFFK (1.15) and Ag9/MFFK (2.31) membranes. This behavior indicates the potential advantage of the developed membranes during long-term operation in a membrane contactor: upon exposure to the mixture being separated, the ethylene transfer will be additionally intensified, which will lead to an increase in separation efficiency.

### 3.3. Ethylene/Ethane Separation in Membrane Contactor Gas-Liquid

**Figure 10:**
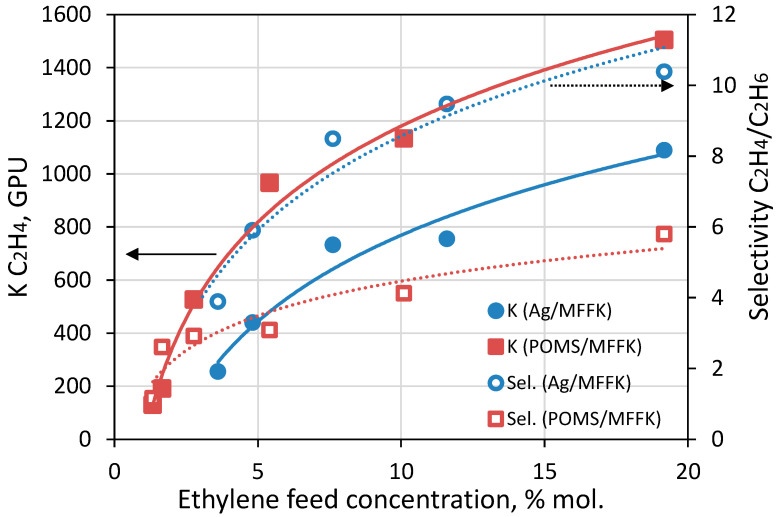
Dependence of the ethylene mass transfer coefficient (K, filled shapes) and ethylene/ethane selectivity (hollow shapes) on the initial ethylene concentration in the mixture for Ag9/MFFK (blue) and POMS/MFFK (red) composite membranes.

A study of the separation of a binary mixture of ethylene-ethane in the process of membrane absorption was carried out on a laboratory gas-liquid membrane contactor. During the project, for the first time, experiments were carried out on the release of ethylene from a mixture in a flat and hollow fiber module, based on membranes with a selective layer of POMS and POMS-Ag9. In the course of an experiment on ethylene release in a gas-liquid membrane contactor, the flat composite membranes Ag9/MFFK and POMS/MFFK were compared. Figure 10 shows the change in the ethylene mass transfer coefficient and ethylene/ethane selectivity, depending on the initial concentration of ethylene in the mixture, at a concentration of silver nitrate in the absorption solution of 3.5 M, a gas supply rate of 3.2 mL/s, and a liquid supply rate of 4.9 mL/s. The general trend is a decrease in mass transfer and selectivity, with a decrease in the concentration of ethylene in the initial mixture. This is not surprising, since the mass transfer coefficient depends on the concentration [49].

In this case, the qualitative difference in the separating ability of Ag9/MFFK and POMS/MFFK membranes seems to be of interest. For the POMS/MFFK membrane, when the initial concentration of ethylene is reduced from 20% to 5%, the mass transfer coefficient of ethylene decreases from 1500 GPU to 970 GPU, and the degree of ethylene recovery is in the range of 45–48%. For the Ag9/MFFK membrane, when the initial concentration of ethylene decreases from 20% to 5%, the ethylene mass transfer coefficient decreases from 1090 GPU to 730 GPU, and the degree of ethylene release is in the range of 35–40%. The lower mass transfer of Ag9/MFFK compared to POMS/MFFK is associated with both the greater thickness of the selective membrane layer and the lower permeability of the membrane material itself. However, the Ag9/MFFK membrane demonstrated higher ethylene selectivity compared to POMS/MFFK. This result was predictable based on the sorption and gas permeability data. Each experimental point was taken after 1 h of contactor operation and, considering the data obtained, it can be argued that the activation of silver nanoparticles has occurred. The increase in the selectivity of ethylene release in the membrane contactor by more than 80% is directly related to the ethylene/ethane selectivity increase of POMS after the introduction of silver nanoparticles into its matrix. The ethylene/ethane selectivity ratio for POMS and POMS Ag membranes demonstrates an improvement in the membrane’s ability to selectively release ethylene. With an increase in the concentration of ethylene in the mixture to be separated, this ratio increases from 1.8 for 20% vol. of ethylene to 3.4 for 4% vol. Thus, it can be said that the introduction of silver nanoparticles into the POMS matrix makes it possible to increase the selectivity of the membrane with respect to ethylene by at least 1.5 times.

Taking into account the positive effect of silver nanoparticles in the POMS polymer matrix on the operation of the gas-liquid membrane contactor during the separation of an ethylene/ethane mixture, membranes with a selective layer based on POMS-Ag9 were chosen for future work.

## 4. Conclusions

For the first time, a method for obtaining membranes POMS-Ag mixed matrix membranes was proposed. It was shown that the dispersion of silver in the POMS matrix is more uniform in the case of using it as a precursor AgBF_4_ in comparison with AgNO_3_. Thus, POMS-Ag mixed matrix membranes with a silver concentration of 0.5 wt% and 9 wt% were obtained. The presence of silver in the form of nanoparticles with a crystallite size of 8 nm was confirmed by X-ray fluorescence analysis, IR-spectroscopy, and WAXD. The effect of silver nanoparticles on the separating ability of membranes was determined by measuring the sorption of ethane, ethylene, and water vapor, as well as during the gas separation of a mixture of ethylene (20% vol.) and ethane (80% vol.). The initial POMS did not have selectivity for ethylene: the sorption selectivity was 0.64 ± 0.02 and the permeability selectivity was 0.85 ± 0.04. The introduction of silver nanoparticles led to an increase in selectivity. With the introduction of 9 wt% nanoparticles into the POMS matrix, selectivity inversion occurred: the average sorption selectivity was 2.5 ± 0.2, while the permeability selectivity was 1.31 ± 0.05. Moreover, it was demonstrated that the exposure of POMS-Ag membranes to the gas mixture being separated for 3 h leads to an increase in ethylene/ethane selectivity: for POMS-Ag1 from 1.0 to 1.3, for POMS-Ag10 from 1.3 to 2.15. Thus, in the obtained POMS-Ag mixed matrix membranes, silver nanoparticles acted as sorption centers for ethylene and had a decisive effect on the separating ability of the membrane. It should be noted that the introduction of silver nanoparticles led to membrane permeability. The introduction of diffusion-impermeable silver nanoparticles led to an increase in the diffusion path of gas molecules. Nevertheless, the permeability of ethylene after exposure of the membranes to the flow of the mixture being separated increased and reached values close to the permeability of POMS.

The comparison of POMS/MFFK and Ag9/MFFK membranes in a gas-liquid membrane contactor demonstrated that the selection of an Ag9/MFFK membrane made it possible to increase the efficiency of ethylene recovery. Therefore, the mass transfer coefficient for ethylene (normalized to the membrane thickness) increased by 20%, and the selectivity of ethylene release doubled when we introduced 9 wt% Ag nanoparticles into the matrix of POMS. Thus, the developed Ag9/MFFK membranes have a great potential for application in gas-liquid membrane contactors.

## Figures and Tables

**Table 1:** Elemental composition of the studied samples of membrane materials.

Symbol	Mass Concentration of AgBF_4_ in the Polymer, wt%	Si Content, wt%	Ag, Content wt%
POMS	0	46.59 ± 0.01	0
POMS-Ag1	1.8	46.37 ± 0.46	0.50 ± 0.11
POMS-Ag9	20	42.0 ± 0.8	9.4 ± 0.6

**Table 2:** Sorption selectivity for the ethylene/ethane, depending on the pressure in the obtained membrane materials.

Pressure, mbar	Sorption Selectivity		
	POMS	POMS-Ag1	POMS-Ag9
45	0.66	0.90	1.73
100	0.64	0.93	2.37
200	0.62	0.77	2.44
400	0.65	0.73	2.29
600	0.64	0.70	2.45
800	0.64	0.68	2.53
1000	0.64	0.65	2.61

**Table 3:** Gas transport properties of POMS/MFFK, Ag1/MFFK, and Ag9/MFFK membranes in the separation of a binary ethylene/ethane mixture before and after exposure to a gas mixture flow.

Membrane	Silver Content, wt%	P/l (Ethane), m^3^/(m^2^atm h)		P/l (Ethylene), m^3^/(m^2^atm h)		Selectivity Ethylene/Ethane	
		0 h	3 h	0 h	3 h	0 h	3 h
POMS/MFFK	0	0.41	0.41	0.35	0.35	0.85	0.85
Ag1/MFFK	0.5	0.20	0.20	0.20	0.23	1.00	1.15
Ag9/MFFK	9.0	0.13	0.13	0.17	0.30	1.31	2.31

## Data Availability

The data presented in this study are available on request from the corresponding author.
